# Implications of Data Extraction and Processing of Electronic Health Records for Epidemiological Research: Observational Study

**DOI:** 10.2196/64628

**Published:** 2025-06-11

**Authors:** Melissa H J van Essen, Robin Twickler, Yvette M Weesie, Ilgin G Arslan, Feikje Groenhof, Lilian L Peters, Isabelle Bos, Robert A Verheij

**Affiliations:** 1 Tranzo, School of Social Sciences and Behavioural Research, Tilburg University, Reitse Poort 1, RP126, Professor Cobbenhagenlaan 125, Tilburg, The Netherlands, 31 631978419; 2 Nivel, Netherlands Institute for Health Services Research, Utrecht, The Netherlands; 3 Department of General Practice and Elderly Care Medicine, University Medical Centre Groningen, Groningen, The Netherlands; 4 National Health Care Institute, Diemen, The Netherlands

**Keywords:** routine health care data, electronic health records, general practice, data governance, data processing, data quality, fitness for purpose, data extraction, ETL, extraction, transformation, and loading

## Abstract

**Background:**

The use of routinely recorded electronic health record (EHR) data is increasingly common, especially in epidemiological research. However, data must be processed and prepared for secondary use, and decisions made during this process could significantly impact research outcomes. A demonstration of the extent of these consequences is necessary.

**Objective:**

The aim of this study was to investigate the influence of data processing steps on research outcomes derived from the secondary use of EHR data.

**Methods:**

EHR data from 8 Dutch general practices from 2019 were used. These practices contributed data to 2 research databases: the Academic General Practitioner Development Network registry and the Nivel Primary Care Database. Data were extracted and processed through distinct extraction, transformation, and loading (ETL) pipelines, allowing the evaluation of the impact of different ETL methods by comparing the 2 datasets in three steps: (1) patient demographics, (2) epidemiology of concordant patients, and (3) health service use of patients with 3 diagnoses. A number of similarity indicators, including the number of contacts, regular consultations and visits, prescriptions, and episodes, were compared between the 2 databases. The outcomes were compared by performing paired samples *t* tests using 99% CIs. Prevalence, number of prescriptions, and number of regular consultations and visits per 1000 patient years were calculated and compared for 3 diagnoses (diabetes mellitus, urinary tract infection, and cough). These outcomes were compared using the SD.

**Results:**

Differences were observed between the datasets in the number of enrolled patients (Academic General Practitioner Development Network registry: n=47,517; Nivel Primary Care Database: n=44,247). Despite this, patient demographics were similar. All indicator outcomes of the concordant patients showed significant differences between the databases, that is, the number of contacts, prescriptions, and episodes per patient, and the number of regular consultations and visits. Differences in the indicator outcomes for the 3 diagnosis groups varied greatly in SD, however, none of the differences were deemed significant.

**Conclusions:**

The findings highlight the importance of routine health data users’ awareness of different ETL steps involved. Transparency and shared knowledge about these processes are critical, and making them available for research is necessary. Data processors should share their knowledge regarding their choices, and researchers and policy makers should invest in their knowledge of this type of metadata. Transparency and shared knowledge are particularly important in light of the European Health Data Space and the ever-increasing secondary use of routinely recorded health data. Future research should focus on the role of transparency, joint decision-making, and the minimization of effects of ETL steps, and on the insight into the individual influence of ETL steps on research outcomes. This could stimulate standardized approaches among data processors and researchers, resulting in increased data interoperability.

## Introduction

Secondary use of routine health care data is progressively more common, such as the use of electronic health records (EHRs) for research and policy making [1-4]. EHR data are frequently used to report the incidence, prevalence, and health service use [3,5-10]. For example, during the COVID-19 pandemic, EHR data facilitated the monitoring of disease spread and health service use [11]. In the Netherlands, general practice EHR data play an important role in research and policy making. Additionally, these valuable data can be used for quality improvement goals without imposing any additional administrative burden on health care professionals [12,13]. From a European perspective, there have been significant developments such as the establishment of a European Health Data Space (EHDS), which should facilitate secondary use of routine health care data [14,15]. Overall, much good is expected from the developments taking place regarding routine health care data.

At the same time, there is an ongoing debate about the fitness for the purpose of EHR data for secondary use [1]. Studies have concluded EHR data to be accurate and reliable for the identification of symptoms and diseases, and to be informative of health care consumption rates, suggesting no further verification is needed prior to the secondary use of these data [16,17]. Additionally, studies agree on the value of EHR data and the broad range of purposes that EHR data could be used for [1,13,16,17]. Others caution against the potential introduction of various types of bias, such as selection bias [1,4]. Since the primary design of EHR data is to record individual patient care as part of the health care process, the fitness for the purpose of this data for secondary use requires careful consideration. Recent research emphasizes the importance of the concept of fitness for purpose and fitness for use, respectively: data serving intended decision-making functions and the ability to get the right information, into the right hands at the right time [10,18].

**Figure 1.**
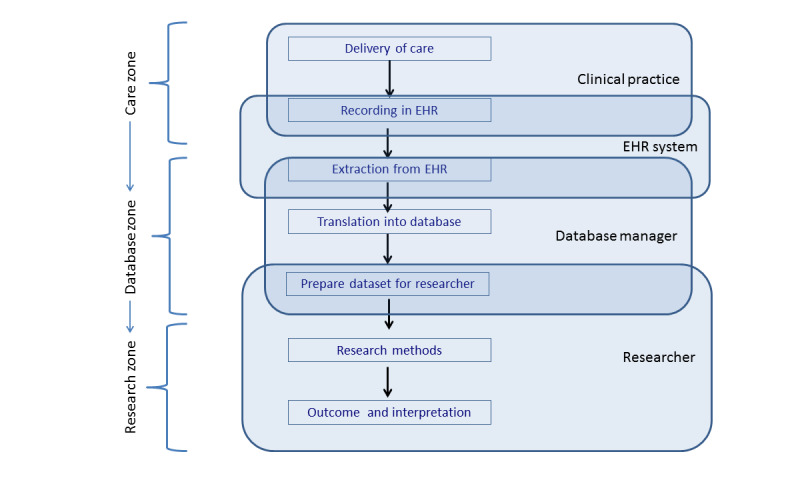
Steps and actors involved in the data flow between the delivery of care and applications reusing the data from Verheij et al [1]. EHR: electronic health record.

Data quality is influenced by factors such as variations in health care professionals’ recording habits caused by high administrative workloads [1,19], inconsistent reporting guidelines, including guidelines for language use in EHR, and variations in EHR software, which can affect morbidity estimates [4,5,20,21]. Additionally, data governance decisions made during the extraction, transformation, and loading (ETL) process—including data preparation and cleaning—contribute to variations in research datasets [1]. Verheij et al [1] visualized the data flow from EHR systems to research datasets into distinct zones (ie, care zone, database zone, and research zone) (Figure 1). Each zone includes the underlying actions (ie, recording in EHR, extracting data, preparing data for research) and actors (ie, physician, database manager, researcher) that contribute to the introduction of potential biases. While efforts have been made to optimize EHR data for secondary purposes and to limit potential bias, such as confounding bias and selection bias, most studies have focused on the completeness of the data, in the “research zone” [5,19,22,23]. Hence, the impact of different ETL methods in the “database zone” on data quality remains underexplored [1,4], leaving stakeholders unaware of potential biases in processing routines.

Therefore, the aim of this study is to investigate the influence of ETL steps involved in the secondary use of general practice EHR data on research outcomes, between 2 different EHR-research databases encompassing data from the same 8 general practices. Using a selection of indicators representative of epidemiological and health services use studies, the difference between the datasets from the 2 respective databases will be investigated. Due to the many differentiating steps within these ETL processes, we expect the indicator outcomes to be different, and to demonstrate the extent of the differences, potentially depending on the extent of the data processing. The results of this study will provide valuable insight into the extent of differentiation between EHR databases and possibly contribute to the awareness of routine health data users of the effects of ETL processes.

## Methods

### Design

This observational study was conducted in the context of the FAIR work packages of 3 larger COVID-19-related collaborations: COVID-GP [2,3,24], Long COVID Mixed Methods [25], and GRIP-3 [26,27]. For these projects, pseudonymized EHR data from general practices were stored and combined on the data platform of Statistics Netherlands. For this study, data from 8 general practices were used, covering the period from January 1 to December 31, 2019. The use of these data allowed for the unique opportunity to evaluate the impact of specific choices made during the different data extraction and processing methods (ie, database zone steps) by comparing the 2 datasets in a three-step approach: (1) patient demographics, (2) epidemiology of concordant patients, and (3) health service use in 3 diagnosis groups.

### Ethical Considerations

Ethical approval for this study was waived by the medical ethics committee of the University Medical Centre Groningen (reference number: 2020/309). The use of EHR data is permitted under certain conditions by Dutch law, both for the data from the general practice registration network (Academic General Practitioner Development Network; AHON) and Nivel Primary Care Database (Nivel-PCD). According to this legislation, neither obtaining informed consent from patients nor approval by a medical ethics committee is obligatory for these types of observational studies that contain no directly identifiable patient data (art. 24 GDPR Implementation Act jo art. 9.2 sub j GDPR). For Nivel-PCD, the project has been approved by the relevant governance bodies of Nivel-PCD under number NZR-00320.087. As mentioned, the EHR data used in this study were pseudonymized before analysis.

### Databases

The datasets used for this study originate from EHR data provided by general practices for 2 research databases: the AHON registry [28] and the Nivel-PCD [29]. The aim of these registries is to provide insights into epidemiology and health care provided in general practices in the Netherlands. The datasets contain pseudonymized EHR data from 56 general practices participating in the AHON registry, located in the North of the Netherlands (approved under number 2020/309) and 363 general practices participating in the Nivel-PCD, located across all regions in the Netherlands (approved under number NZR-00320.087). Eight of these 56 general practices contributed to both databases. The structured data from these shared practices was used to compare research outcomes for the distinctly processed research datasets of the AHON registry and Nivel-PCD.

### Data Extraction and Processing Pipelines

The AHON registry receives EHR data from a third party that extracts and processes the data. Nivel-PCD receives extracted EHR from the EHR system provider of the general practitioner (GP), after pseudonymization by a third party. This process follows extraction specifications formulated by the AHON registry and Nivel-PCD, respectively, a distinct step in the ETL process for the 2 research databases. The translation into the registry and preparation of the dataset for the researchers involves distinct steps and choices as well. Textbox 1 provides an explanation of the differences in processing of each variable between the AHON registry and Nivel-PCD, and the zones in which processing takes place.

Textbox 1. **Variables used for analyses (for the Academic General Practitioner Development Network [AHON] registry and Nivel Primary Care Database [Nivel-PCD]): definitions and processing zones.**

**Care Zone Variables:**

**International Classification of Primary Care (ICPC) Code**

- Diagnosis codes are based on the ICPC-1 coding system. [30] These codes have been recorded in the electronic health record (EHR) by the general practitioner (GP), and are linked to prescriptions, contacts or actions performed by the GP.
- **AHON registry:** ICPC codes as recorded by the GP.
- **Nivel-PCD:** ICPC codes as recorded by the GP.

**Anatomical Therapeutic Chemical (ATC) Code**

- Prescription codes are based on the ATC classification system for the recording of medication. [31] ATC-codes are recorded in the EHR, by either the GP or via feedback from the pharmacist.
- **AHON registry:** ATC-codes as recorded by the GP. No feedback from different health care providers
- **Nivel-PCD:** ATC-codes recorded in the GP system. Originating from recordings of GPs or from feedback provided by pharmacies. Distinguishing between the 2 sources is not feasible.

**Episodes**

- Depending on the EHR system, the end of an episode is automatically recorded in the EHR or this is done manually by the GP. Symptoms or comorbidities can be linked to an episode with the corresponding ICPC code.
- **AHON registry:** Based on the episodes of care as recorded in the EHR.
- **Nivel-PCD:** Not applicable: See “Episode-construct” under Database Zone Variables within this textbox.

**Database Zone Variables:**

**Registration quarter**

- The yearly quarter a patient was enrolled at the general practice, based on capitation fees that are recorded on a quarterly basis for each patient that is enrolled in the practice during that quarter. The datasets in this study contain information on enrolled patients in 2019, and as such the maximum number of registration quarters per patient is 4.
- **AHON registry:** Registration duration is based on the date of enrollment of a patient. Only fully registered quarters are included. When a patient enrolls halfway through the quarter, the registration will start from the next quarter.
- **Nivel-PCD:** Registration duration is determined by capitation fee records. Only patients enrolled for a full quarter are included. Mid-quarter registrations start the following quarter. Missing quarters between first and last registrations are imputed.

**Pseudonymized patient identification number**

- A unique number assigned to each patient in a dataset stored on the data platform of Statistics Netherlands. This pseudonymized identifier allows patient information to be linked to other datasets available on the data platform.
- **AHON registry**: For patients uploaded to the data platform of Statistics Netherlands this is based on 3 numbers of the postal code (PC3), year of birth, and sex of the patient.
- **Nivel-PCD**: Based on the social security number of the patient. For patients uploaded to the data platform of Statistics Netherlands as well as the usual datasets.

**Prescriptions**

- In this study defined as prescribed medications, based on the record of a unique ATC-code on a unique date.
- **AHON registry**: Contains all medications prescribed by the GP. Including repeat prescriptions.
- **Nivel-PCD**: Contains all medications recorded in the EHR prescribed by the GP. Including repeat prescriptions, or by a different health care provider, see “ATC-code” under care zone within this textbox. Prescriptions are deduplicated in case of a double record within 8 days, for example in case of a record by the GP and a record by the pharmacist.

**Insurance claims codes**

- Based on the insurance claims database (Vektis) classification system designed to record and invoice all activities of the GP [32]. It can be further divided into activities during practice consultations, home visits, other contacts, and capitation fee records. Activities can be linked to an ICPC code on the same day by the data processor, and used to classify and invoice actions such as interventions performed in the general practice, thus providing insight into the invoiced activities of a GP. Insurance claims codes are recorded in the care zone, and processed in the database zone.
- **AHON registry:** All insurance claims codes as recorded by the GP are included. Including the code for recording the capitation fees of a patient each quarter.
- **Nivel-PCD:** All insurance claims codes as recorded by the GP are received, however, when a dataset has been requested by the researcher, the codes are filtered by the data processor to include a selection of codes relevant to the study (in agreement with the researchers), on a database zone level.

**Contacts**

- Defined as moments of contact between a GP and a patient. Based on unique dates on which an insurance claim code was recorded by the GP, that is, the maximum number of contacts per patient per day is 1. Insurance claims codes are a classification system designed to record all actions of the GP, and can be further divided into practice consultations, home visits and other contacts. The recording of ICPC codes and ATC-codes can be linked to contacts by the data processor, based on date.
- **AHON registry**: Contains all insurance claims codes present in the general practice EHR system. Including interventions carried out by GP practice support.
- **Nivel-PCD:** Contains a selection of insurance claims codes relevant to the research.

**Episode-construct**

- An adaptation of the recorded episodes of care as recorded in the EHR by the GP. EHR data of the current year and the 2 prior years are used to construct the episode. A diagnosis is labeled an episode of illness from the date of diagnosis to the last encounter plus half of the duration of the contact-free interval. [33]
- **AHON registry:** See “episodes” under care zone within this textbox.
- **Nivel-PCD:** Episodes are based on the episode construct. The Nivel-PCD thus actively enters an “end-date” for certain episodes based on this construct, independently of the recording of the GP. Within this construct, when a symptom, such as coughing, is recorded under an episode such as asthma, the symptom will be overruled by the episode.

**Research Zone Variables:**

**Patient year**

- Duration of the year that a patient was registered at a general practice. Calculated based on registration quarters (1-4), thus the minimum number of patient years per patient is 0.25, and the maximum number of patient years per patient is 1. Patient years are calculated by the researcher.
- **AHON registry:** Operationalization is conducted following the description using registration quarters and pseudonymized patient identification numbers.
- **Nivel-PCD:** Operationalization is conducted following the description using registration quarters and pseudonymized patient identification numbers.

**Prevalence rate**

- The total number of patients with a disease existing in the population, in this study per 1000 patient years. The corresponding formula is the number of patients with the record of the ICPC diagnosis code/number of patient years of the population * 1000. The ICPC codes used for this calculation are contact-ICPC codes. The maximum number of disease cases per ICPC code is 1 per patient.
- **AHON registry:** Operationalization is conducted following the description using ICPC codes and patient years.
- **Nivel-PCD:** Operationalization is conducted following the description using ICPC codes and patient years.

**Regular consultations and visits**

- A subselection of contacts as described under “Database zone.” The subselection is based on insurance claims codes representing regular consultations and visits [32], and is identical for AHON registry and Nivel-PCD, namely:12001 – regular consultation, > 20 minutes12002 – regular visit, < 20 minutes12003 – regular visit, > 20 minutes12010 – regular consultation, < 5 minutes12011 – regular consultation, > 5 minutes < 20 minutes
- **AHON registry:** All subselected insurance claims codes are present in the AHON registry dataset, and no further data processing takes place on these codes.
- **Nivel-PCD:** All subselected insurance claims codes are present in the Nivel-PCD dataset, and no further data processing takes place on these codes.

### Population

**Figure 2.**
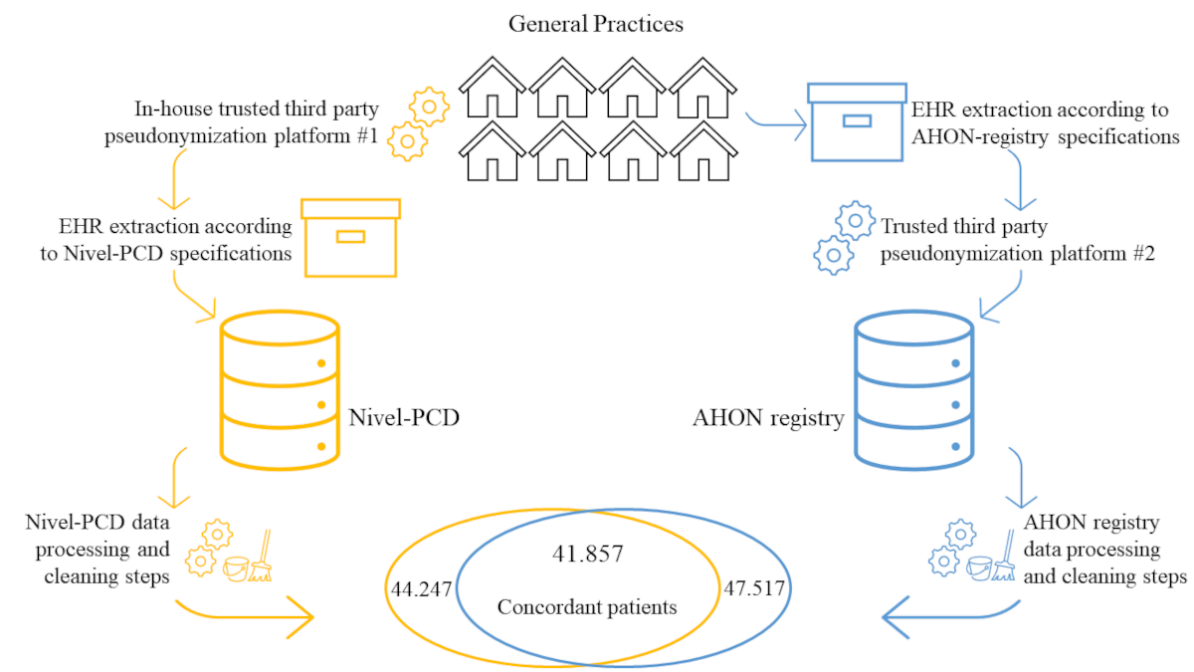
Flowchart of patients included in the study population. AHON: Academic General Practitioner Development Network (Academische Huisartsen Ontwikkel Netwerk); EHR: electronic health record; Nivel-PCD: Nivel Primary Care Database.

The population consisted of all individuals enrolled as patients for at least one quarter in one of the 8 shared general practices. A subgroup of concordant patients was used for part of the analyses. The concordant patient group comprised patients present in both databases (41,857/49,907, 83.9%), based on their identical identification numbers as assigned by Statistics Netherlands. This identification number was derived from a pseudonym of the social security number for patients in the Nivel-PCD and, for patients in the AHON registry, a pseudonym of a combination of 3 digits of the postal code (PC3), year of birth, and sex. The concordant patient group was the focus of the second step of our 3-step approach,—the epidemiology of concordant patients—enabling an accurate assessment of data similarity between patients in the 2 research datasets. Nonconcordant patients were excluded to avoid automatically skewed results. The total group of patients—all those present in the databases—was used for the first and third steps, namely the analyses of patient demographics and health service use in 3 diagnosis groups. This approach minimized selection bias, as research on health service use conducted with EHR data usually does not allow for the filtering of such patients. Figure 2 provides a complete flowchart of the population inclusion.

### Indicators of Similarity

The datasets include data on patient demographics, including age, sex, postal code, and registration quarter, as well as information on the number, type, and reason of contacts, including consultations, visits, and other interventions. Diagnoses or symptoms, along with the number of and indications for prescriptions on patient-level, were included as well. Information on diagnoses included the International Classification of Primary Care (ICPC)-1 codes and information on prescriptions included the Anatomical Therapeutic Chemical codes [30,31]. For consultations, visits, and other interventions, the insurance claims codes were included, which were used to record and invoice all activities of the GP, along with corresponding dates. These data are provided in different modules as follows: patient table, contacts table, interventions table, and prescriptions table. Each variable within these tables represents a record by the GP.

**Figure 3.**
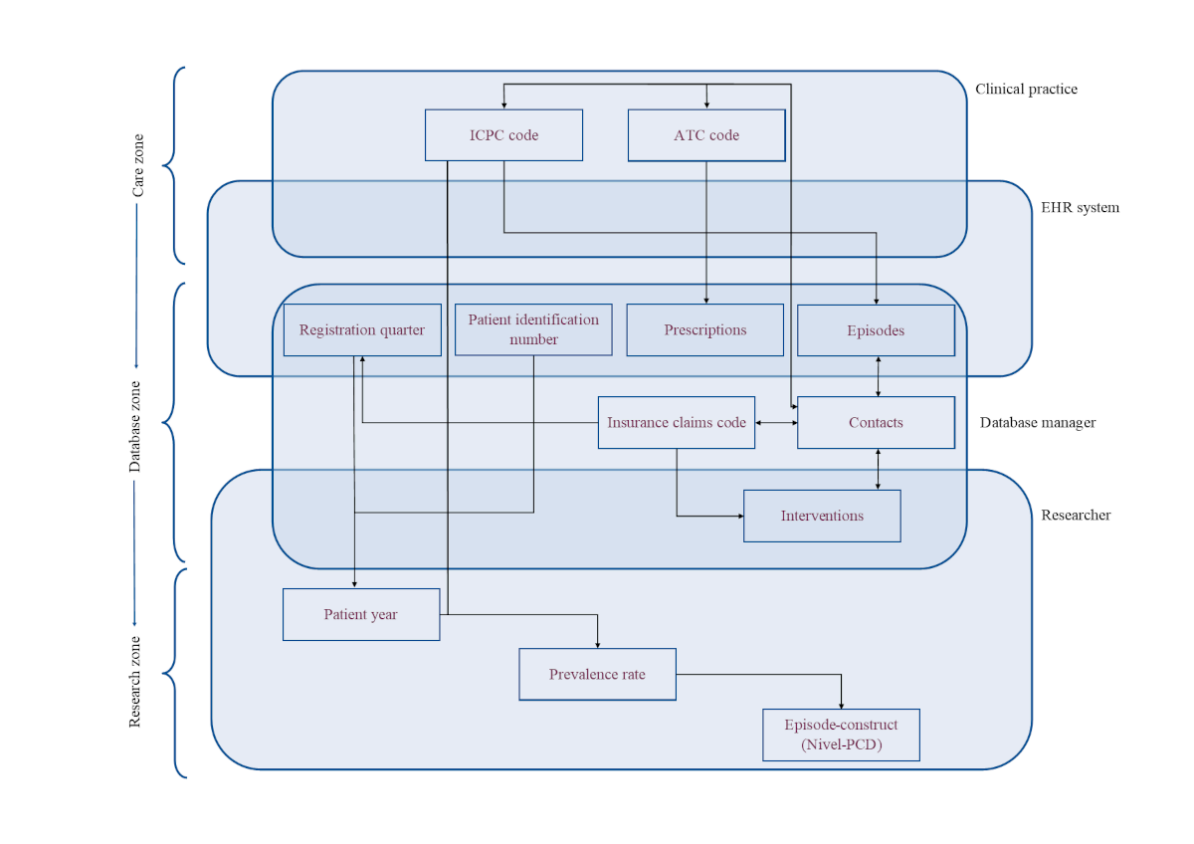
Schematic overview of the variables and their relationship, and origin. ATC: Anatomical Therapeutic Chemical; EHR: electronic health record; ICPC: International Classification of Primary Care; Nivel-PCD: Nivel Primary Care Database.

Indicators were operationalized differently in each database based on requirements set by the principal investigators of the main project as preparation for the researchers (Figure 1). Definitions and operationalization of variables or records, as well as the “zone” in which the records were processed, are detailed in Textbox 1. When records were processed differently, an explanation of the processing step is included for the relevant database, that is, AHON registry or Nivel-PCD. Variables from the “research zone” (Textbox 1), such as patient years, prevalence rate, and regular consultations and visits, were operationalized in identical ways, as explained in Textbox 1. Figure 3 provides a schematic overview of the connections between the variables and the processing zones. Variables are placed in blue fields representing the actor responsible for producing or processing them, with arrows indicating the relationship between the variables. The further down a variable is placed in the overview, the more that variable has been processed.

### Analyses

#### Overview

Demographic analyses, analyses on the epidemiology of concordant patients, and analyses on the health service use of patients in 3 diagnosis groups were performed on the Statistics Netherlands remote access platform, where both datasets were uploaded and stored.

#### Step 1: Demographics

Demographic analyses were performed on all patients present in the databases. The total number of unique patients based on the identification number, the total number of patient years, the mean number of patients per practice, the number of patients by age category (0‐4, 5‐17, 18‐64, and 65+ y), and sex were calculated. These demographic analyses provided insights into potential differences between the 2 populations and contextualized a relevant perspective on the outcomes of 3, the health service use analyses in 3 diagnosis groups. Due to the differences in pseudonymization methods and registration quarters processing methods, a minor difference in the demographics of the AHON study population and the Nivel-PCD study population was anticipated. For a definition of patient years and all other variables used for the analyses, see Textbox 1.

#### Step 2: Epidemiology of Concordant Patients

To identify differences present on a patient level, we analyzed the similarity of data present between the 2 datasets for the concordant patient group. The use of the concordant patient group ensured that detection bias was minimized and differences in outcome measures present were not overestimated due to population discrepancies. We compared the mean and SD of similarity indicators present in both datasets at the patient level: the number of contacts, number of regular consultations and visits, number of prescriptions, and number of episodes. For these analyses, we merged the available indicators based on the mutual identification number of the patient and compared the mean and SD for each indicator by performing paired samples *t* tests and calculating the corresponding 99% CIs, that is, the confidence level was set to 99% due to the large number of data. A larger difference was expected in the number of contacts and episodes, due to the more extensive data processing that took place for these variables (Textbox 1), especially due to the presence of the episode construct for Nivel-PCD episodes. For the number of regular consultations and visits, we expected no significant differences as the data extraction and processing on these insurance claims codes were largely identical for the 2 databases. Analyzing the health care consumption of patients based on a subselection of relevant insurance claims codes, as opposed to a nonspecific selection, that is, all insurance claims codes, is more representative of research conducted with EHR data, as this is a more commonly used method among researchers.

#### Step 3: Analyses of the Health Service Use in 3 Diagnosis Groups

Subsequently, a set of indicators was selected to provide a way to compare the research outcomes of both datasets for different diagnoses. The purpose of these analyses is to observe the possible effects of the different data ETL pipelines to which the 2 datasets have been subjected. Hence, the analyses were performed for all patients present in the datasets.

### Selection of Indicators

The indicators were selected by a research team with expertise in research conducted with routine health care data, and data processors of the 2 databases. With this set of indicators, we aimed to be representative of the research that is typically conducted with these datasets [3,28,29,34]. When selecting the set of indicators, the selection process focused on including the diagnosis of a chronic or long-term condition, an acute condition, and a symptom with a high prevalence within Dutch general practices and a high disease burden. Additionally, we were careful to ensure that the selection of indicators used all available data provided by the databases, to ensure that any relevant potential differences present in the data were detected in the outcomes. We included patients with diabetes mellitus (DM), urinary tract infection (UTI), and cough diagnosis. For each of these diagnosis groups, we calculated the total number of patients by including those with a relevant ICPC code recorded in the EHR: T90 for DM, U71 for UTI, and R05 for cough. The prevalence rate per 1000 patient years was calculated by dividing the number of patients with one of these ICPC diagnosis codes recorded by the number of patient years of the population, then multiplying by 1000. Additionally, we calculated the number of regular consultations and visits these patients received per 1000 patient years, and the number of prescriptions per 1000 patient years. This was done by selecting a group of Anatomical Therapeutic Chemical codes for each diagnosis, as indicated by the pharmacotherapeutic compass, a Dutch web-based reference book that provides independent pharmaceutical information for medical professionals: A10A and A10B for DM; G03C, J01C, J01D, J01E, J01G, J01M, and J01X for UTI; and R05C, R05D, R05X, and R06A for cough [35].

For each diagnosis group, the differences in the indicators were compared using the SD, meaning the proportion of patients based on the AHON registry was compared with the proportion of people based on Nivel-PCD:

$SD=\frac{{\hat{p}}_{\text{AHON}}-{\hat{p}}_{\text{NPCD}}}{\sqrt{\frac{{\hat{p}}_{\text{AHON}}(1-{\hat{p}}_{\text{AHON}})+{\hat{p}}_{\text{NPCD}}(1-{\hat{p}}_{\text{NPCD}})}{2}}}$

Where AHON=AHON registry and NPCD=Nivel-PCD. According to Austin [36], absolute values of the SD of 0.2, 0.5, and 0.8 correspond to small, medium, and large differences, respectively [37]. Remaining cautious to some extent, and in order to disclose small differences, differences with absolute values of the SD>0.2 were considered to be significant.

All analyses were performed using R (version 4.2.3; R Foundation for Statistical Computing) and RStudio (version 2022.02.1+461 “Prairie Trillium”; R Foundation for Statistical Computing). For definitions of patient years, prevalence rate, and prescriptions, see Textbox 1.

## Results

### Population and Demographics Characteristics

This study included all patients who were registered for at least one quarter in 2019 at 1 of 8 general practices represented in both the AHON registry and the Nivel-PCD. This resulted in a total of 49,907 patients, of which 47,517 patients were present in the AHON registry database and 44,247 patients were present in the Nivel-PCD. A subgroup of 41,857 patients present in both datasets, referred to as “concordant patients,” was identified. The total number of patients in the general practices prior to data extraction from the EHR system was unavailable.

Table 1 provides an overview of the demographic characteristics of all patients combined. The patient demographics, age category, and sex were similar in both datasets. There was a difference in the total number of unique patients (n=47,517 vs n=44,247), the number of patient years (n=46,400 vs n=43,100), and the mean number of patients per practice (n=5940 vs n=5531). The latter difference was largely influenced by one outlier practice. We found no statistically significant differences in the demographic characteristics between the datasets, with all *P* values >0.5.

**Table 1.** Demographic characteristics of the study population (all patients, N=49,907).

Characteristic	AHON^a^ registry (n=47,517)	Nivel-PCD^b^ (n=44,247)
Patient years	46,400	43,100
Patient years per patient	0.977	0.974
Patients per practice, mean (SD)	5940 (1313.3)^c^	5531 (1803.4)^c^
Age category (years), n (%)		
0‐4	2239 (4.7)	2093 (4.7)
5‐17	7382 (15.5)	6917 (15.6)
18‐64	27,507 (57.9)	25,684 (58.0)
65+	10,389 (21.9)	9553 (21.6)
Sex, n (%)		
Male	23,471 (49.2)	21,968 (49.6)
Female	24,046 (50.6)	22,279 (50.4)

a AHON: Academic General Practitioner Development Network.

b Nivel-PCD: Nivel Primary Care Database.

c Difference in mean number of patients per practice largely due to one outlier.

### Similarity in Epidemiology of Concordant Patients

The number of contacts, regular consultations and visits, prescriptions, and episodes per patient were analyzed for the concordant patients in each dataset. By comparing these outcomes, statistically significant differences were obtained between the AHON registry and Nivel-PCD. All differences were significant. In the AHON registry, the mean number of contacts recorded per patient was 8.58 (SD 10.10), while in the Nivel-PCD this average was 7.40 (SD 9.02). The average number of regular consultations and home visits per patient was 4.33 (SD 5.67) in the AHON registry and 4.30 (SD 5.65) in the Nivel-PCD. The number of prescriptions was higher in the AHON registry, with an average of 6.75 (SD 11.30) per patient, compared with 5.90 (SD 9.45) in Nivel-PCD (*P*<.001). The number of episodes was significantly lower for the patients in the AHON registry: 1.61 (SD 1.73) episodes per patient in 2019, while patients in the Nivel-PCD had a mean number of 3.74 (SD 3.67) episodes in 2019 (*P*<.001). Table 2 provides detailed outcomes for all indicators of similarity in the concordant patient group.

**Table 2.** Epidemiology of the concordant study population.

Outcome	AHON^a^ registry (n=41.857), mean (SD)	Nivel-PCD^b^ (n=41.857), mean (SD)	Statistical differences between AHON registry and Nivel-PCD	
			*P* value	95% CI^c^
Number of contacts per patient	8.58 (10.10)	7.40 (9.02)	<.001	1.17 to 0.01
Number of regular consultations and visits per patient	4.33 (5.67)	4.30 (5.65)	.46	–0.07 to 0.13
Number of prescriptions per patient	6.75 (11.30)	5.90 (9.45)	<.001	0.83 to 0.87
Number of episodes per patient	1.61 (1.73)	3.74 (3.67)	<.001	2.11 to 2.15

a AHON: Academic General Practitioner Development Network.

b Nivel-PCD: Nivel Primary Care Database.

c 95% CI of the mean difference.

### Analyses on Health Service Use in 3 Diagnosis Groups

In step 3, we analyzed the prevalence rate, number of prescriptions, and the number of regular consultations and visits per 1000 patient years for 3 different diagnosis groups: DM, UTI, and cough. The differences varied greatly, but none were deemed significant. There were no significant differences in the prevalence rates for the DM (SD −0.01), UTI (SD −0.10), and cough (SD 0.19) diagnosis groups, as shown in Table 3. The SD between the number of prescriptions per 1000 patient years was 0.12 for the DM diagnosis group, 0.01 for the UTI diagnosis group, and −0.001 for the cough diagnosis group. The number of regular consultations and visits did not significantly differ across the 3 diagnosis groups either, with SDs of −0.05 for the DM group, −0.19 for the UTI group, and −0.18 for the cough group. All SDs are presented in Table 3.

**Table 3.** Indicator outcomes for 3 diagnoses.

Indicator	AHON^a^ registry (n=47,517) (patient years=46,400)	Nivel-PCD^b^ (n=44,247) (patient years=43,100)	SD between AHON registry and Nivel-PCD
Diabetes mellitus (T90) diagnosis			
Number of patients with ICPC^c^ T90 record	2979	2787	N/A^d^
Patient years	2936	2728	
Patient years per patient	0.986	0.979	
Prevalence rate	64.2	64.7	−0.01
Number of prescriptions per 1000 patient years	447.0	377.4	0.12
Number of regular consultations and visits per 1000 patient years	498.1	529.6	−0.05
Urinary tract infection (U71) diagnosis			
Number of patients with ICPC U71 record	2960	3005	N/A
Patient years	2927.25	2961.25	
Patient years per patient	0.977	0.977	
Prevalence rate	63.8	69.7	−0.10
Number of prescriptions per 1000 patient years	138.1	135.0	0.01
Number of regular consultations and visits per 1000 patient years	722.1	823.3	−0.19
Cough (R05) diagnosis			
Number of patients with ICPC R05 record (patient years, patient years per patient)	2404	2718	N/A
Patient years	2356.5	2663.25	
Patient years per patient	0.980	0.980	
Prevalence rate	51.8	63.1	−0.19
Number of prescriptions per 1000 patient years	50.8	51.0	−0.001
Number of regular consultations and visits per 1000 patient years	426.4	536.7	−0.18

a AHON: Academic General Practitioner Development Network.

b Nivel-PCD: Nivel Primary Care Database.

c ICPC: International Classification of Primary Care.

d N/A: not applicable.

## Discussion

### Principal Findings

This study investigated the influence of data extraction and processing on research outcomes derived from routine health care data by comparing indicators of similarity based on EHR data from 8 general practices processed through 2 different ETL methods. Despite the identical origin of the data, differences in data extraction and processing pipelines, as well as the choices made by several actors (eg, data processors and researchers), during different stages of these processing methods by each database, resulted in different indicator outcomes. Our results show more substantial differences when data has been more extensively processed, and no significant differences when processing was minimized.

The value of EHR data is increasingly recognized, along with the acknowledgment of the need for reliable and valid data. However, our findings emphasize the need for transparency regarding the data governance and the motives behind the ETL steps, as well as adequate metadata to document these decisions [38,39]. For example, these choices often originate from the inclination to improve the validity of the outcomes. Moreover, interoperability challenges extend beyond uniform EHR systems or standardized coding or ontologies [38,40].

The results support the expectation that ETL choices can significantly affect research outcomes. This underscores the relevance of adopting transparency in the approach to obtaining, processing, analyzing, but moreover interpreting the data, when aiming for appropriate quality. Additionally, it shows the complexity of data processing and the coordination of certain definitions of variables between data processors and researchers. Furthermore, researchers conducting future studies with EHR data should be mindful of data processing choices made, and data processors should share their knowledge about these choices. Additionally, users of EHR data, such as researchers and policy makers, should invest in their knowledge of metadata, as transparency is becoming increasingly critical in the context of developments such as the EHDS.

### Principal Results

First, we compared the demographic characteristics of all patients in the study population and noted differences in the number of unique patients, patient years, and the mean number of patients per practice. This may be explained by the different pseudonymization methods, as the AHON registry uses the PC3 postal code, year of birth, sex of the patient, and the Nivel-PCD uses the pseudonym of the social security number of the patient. After pseudonymization, patient data were uploaded, stored, and combined on the data platform of Statistics Netherlands. Previous research shows that similar procedures result in a loss of coverage of linked patients [41]. The difference in patient years may be caused by the data processing of the registration quarters in each database, as each databases’ patient years are based on differentiating dates of registration quarters, and the occurrence of imputation of registration quarters takes place for Nivel-PCD only.

Second, we compared the epidemiology of the concordant patient group and found significant differences between the AHON registry and Nivel-PCD in the average number of contacts, prescriptions, episodes, and regular consultations and visits per patient. This implies that differences occur on the level of the datasets as a whole, as seen from the demographic analysis results, as well as for the exact concordant patients. We conclude these differences occur due to different data ETL methods, and additionally, due to the impact of choices on the analyses. For example, a specific selection of insurance claims codes for regular consultations and visits, although significantly different, results in a similar mean number per patient, implying that thorough coordination of variables may mitigate the effects of different data processing methods. Nivel-PCD and AHON registries apply different exclusion steps for insurance claims codes in preparation for a research dataset, resulting in a large difference in the total number of insurance claims codes and the type of codes included. The subselection of these claims codes for regular consultations and visits was specifically coordinated, resulting in no significant difference in the number of regular consultations and visits between the 2 databases. When identical definitions are used for regular consultations and visits, and minimal processing steps have taken place on the variables used, no significant differences are observed between the 2 databases. This highlights the importance of clear variables and ETL specification when analyzing data [39]. Interpretation differences can occur when insurance claims codes are analyzed in general. As data are increasingly being shared, for example between countries, researchers with limited knowledge of certain health care systems or data processing methods can unintentionally present biased results. Meta-information on data extraction and processing choices, as well as research methods, could be a solution. The difference in number of episodes may be attributed to the Nivel-PCD episode construct. The Nivel-PCD episode construct algorithm takes all episodes recorded by GPs into account, as well as contacts with the GP and prescriptions as recorded in the EHR. Additionally, this construct includes episodes that were started at the end of the previous year and continue into the next year [33]. For AHON registry diagnoses based on the episode ICPC, these episodes are not included. The episode construct, hence, possibly increases the number of episodes compared with the number of episodes based on general practice records. This methodological distinction aligns with the results of this study.

Lastly, we investigated the effects of the different data extraction and processing methods on a selection of indicator outcomes, comparable to real-world research conducted with these datasets, by analyzing the health service use in 3 diagnosis groups. For these indicators, we found no SDs that were deemed significant. The prevalence rates for the UTI diagnosis group and the cough diagnosis group showed greater differences, while the prevalence rates for the DM diagnosis group did not. This may be attributed to the processing that takes place for ICPC codes within the contacts table, which includes the ICPC codes of patients who have visited the GP for this specific diagnosis. Patients with chronic illnesses may visit the GP more frequently for their diagnosis compared with patients with an acute illness, potentially diminishing the effect of ETL differences, as the maximum number of disease cases for the prevalence rate was one per patient. Additionally, differences may be attributed to the differences in the processing of patient years and the pseudonymization process.

The variance in the SD among prevalence rates and the additional indicators, along with the discrepancy of significant differences for the concordant patient group and no significant differences for the 3 diagnosis groups, shows that the severity of the differences may range from irrelevant to significant. This may be conditional on the purpose of the use of these data. In this study, the results of the indicators are dependent on the dataset that is used to answer the research questions, as well as the method to analyze these data. The interpretation of these outcomes is relevant because research outcomes are often used for purposes such as policy making and feedback information to health care professionals, and the approach of interpreting research outcomes when handling imperfect data is thus consequential. Third parties using EHR data for secondary uses should therefore not dismiss the value EHR data has to offer, such as the broadness of research outcomes available, as demonstrated in this study, but rather focus on improving the manner in which these data are handled. The criticality of the interpretation of research outcomes may be different for research outcomes based on trends over time. In other words, when the data extraction and processing methods remain identical for several datasets over the years, and data processors and researchers focus on data robustness, outcome measures on trends over time might remain reliable. This should be explored in future research.

### Comparison With Prior Work

The observed differences in outcomes of the indicators across all 3 steps highlight the necessity of transparency and joint decision-making with the knowledge of researchers on the dataset that is being used. Instructions on the fitness for purpose and the data quality can be included in the documentation, and clear communication between data processors and researchers is crucial for the interpretation of researchers and policy makers on the results of their study. The outcomes of this study suggest that frameworks to improve fitness for purpose could be a necessary tool in analyzing and interpreting the data. Previous research has resulted in a data quality assessment framework to improve the quality of the datasets that are used for secondary purposes such as research, but it does not elaborate on the effects that processing steps can have on research outcomes [42].

Differences in outcomes that occur due to data processing emphasize the need to make joint decisions regarding ETL pipelines, as this may increase interoperability, for example, between research databases. To achieve this, documentation regarding this process is essential, and the need for detailed meta-information is crucial in this type of research. Interoperability also requires collaboration within and between data processors and researchers [43]. This cooperation will lead to better interpretations of the research conducted with these types of data, and previous research concludes there are benefits to be gained from research on optimal common standards [44]. Similar common approaches have been recommended to improve data quality and have resulted in a harmonized data quality assessment framework [45]. To stimulate interoperability and increase data quality [46], frameworks such as these should become common practice before analyzing EHR data.

### Limitations

A limitation of this study is the choice of diagnoses (ie, DM, UTI, and cough) with high prevalence rates, possibly making the indicator outcomes less applicable to smaller diagnostic groups, for example, of rare conditions [47]. These diagnoses were selected to ensure sufficient patient numbers per diagnosis as the number of concordant general practices in the databases was small. An additional limitation is the lack of detailed information on the data ETL steps that were taken for each database. This was due to the fact that this study started with the end products, that is, the research datasets, as opposed to the unprocessed data coming directly from the EHR systems. Despite the limitations, this data was prepared for research, irrespective of this study, which decreases bias in the preparation methods of the extraction and data processing for these research datasets. Moreover, this study appears to be the first to compare data processing methods with concordant general practices and hence contributes to gaining insight into the influence of these methods on research outcomes based on EHR data.

### Conclusions

In conclusion, routine health care data such as EHR data from general practices offer a broad spectrum of applications, and the secondary use of these data is ever-increasing. Moreover, the results show the impact of data processing steps and analysis choices on the selected indicators and the necessity of transparency between the knowledge of data processors regarding these choices and the knowledge of researchers of this type of metadata. Researchers and policy makers should be cautious with the secondary use of EHR data, especially with regard to the interpretation of research outcomes. Future research should focus on this transparency and the benefits of using a data quality framework intended to minimize the effects of data processing steps, and on gaining more insight into the individual influence of different processing steps on different research outcomes. This could stimulate a common approach among data processors and researchers and thus increase interoperability, which is all the more important with regard to developments such as EHDS and the ever-increasing secondary use of routinely recorded health data.

## Abbreviations

AHON: Academic General Practitioner Development Network (Academische Huisartsen Ontwikkel Netwerk)
DM: diabetes mellitus
EHDS: European Health Data Space
EHR: electronic health record
ETL: extraction, transformation, and loading
GP: general practitioner
ICPC: International Classification of Primary Care
Nivel-PCD: Nivel Primary Care Database
UTI: urinary tract infection

## References

[1] Verheij RA, Curcin V, Delaney BC, McGilchrist MM (2018). Possible sources of bias in primary care electronic health record data use and reuse. J Med Internet Res.

[2] Ramerman L, Rijpkema C, Bos N, Flinterman LE, Verheij RA (2022). The use of out-of-hours primary care during the first year of the COVID-19 pandemic. BMC Health Serv Res.

[3] Rijpkema C, Ramerman L, Homburg M (2023). Care by general practitioners for patients with asthma or COPD during the COVID-19 pandemic. NPJ Prim Care Respir Med.

[4] Grath-Lone LM, Jay MA, Blackburn R (2022). What makes administrative data “research-ready”? A systematic review and thematic analysis of published literature. Int J Popul Data Sci.

[5] Arslan IG, Damen J, de Wilde M (2022). Incidence and prevalence of knee osteoarthritis using codified and narrative data from electronic health records: a population-based study. Arthritis Care Res (Hoboken).

[6] Violán C, Foguet-Boreu Q, Hermosilla-Pérez E (2013). Comparison of the information provided by electronic health records data and a population health survey to estimate prevalence of selected health conditions and multimorbidity. BMC Public Health.

[7] (2024). How we collect data. Institute for Health Metrics and Evaluation.

[8] (2024). Health service data. World Health Organization.

[9] Poos M, Gommer A, Nielen M (2024). Gebruik huisartsenregistraties voor schattingen morbiditeit [use of GP registrations for morbidity estimates]. VZinfo website.

[10] Vanhommerig JW, Verheij RA, Hek K (2025). Data resource profile: nivel primary care database (Nivel-PCD), the Netherlands. Int J Epidemiol.

[11] Madhavan S, Bastarache L, Brown JS (2021). Use of electronic health records to support a public health response to the COVID-19 pandemic in the United States: a perspective from 15 academic medical centers. J Am Med Inform Assoc.

[12] Horrocks S, Wilkinson T, Schnier C (2017). Accuracy of routinely-collected healthcare data for identifying motor neurone disease cases: a systematic review. PLoS ONE.

[13] Dash S, Shakyawar SK, Sharma M, Kaushik S (2019). Big data in healthcare: management, analysis and future prospects. J Big Data.

[14] (2024). What is the European Health Data Space (EHDS)?. The European Health Data Space (EHDS).

[15] Marcus JS, Martens B, Carugati C, Bucher A, Godlovitch I (2022). The European Health Data Space.

[16] Harper C, Mafham M, Herrington W (2021). Comparison of the accuracy and completeness of records of serious vascular events in routinely collected data vs clinical trial-adjudicated direct follow-up data in the UK: secondary analysis of the ASCEND randomized clinical trial. JAMA Netw Open.

[17] Ta CN, Dumontier M, Hripcsak G, Tatonetti NP, Weng C (2018). Columbia open health data, clinical concept prevalence and co-occurrence from electronic health records. Sci Data.

[18] Barbazza E, Klazinga NS, Kringos DS (2021). Exploring the actionability of healthcare performance indicators for quality of care: a qualitative analysis of the literature, expert opinion and user experience. BMJ Qual Saf.

[19] Månsson J, Nilsson G, Björkelund C, Strender LE (2004). Collection and retrieval of structured clinical data from electronic patient records in general practice. a first-phase study to create a health care database for research and quality assessment. Scand J Prim Health Care.

[20] Arslan IG, Damen J, de Wilde M (2022). Estimating incidence and prevalence of hip osteoarthritis using electronic health records: a population-based cohort study. Osteoarthr Cartil.

[21] van den Dungen C, Hoeymans N, van den Akker M (2014). Do practice characteristics explain differences in morbidity estimates between electronic health record based general practice registration networks?. BMC Fam Pract.

[22] Haneuse S, Daniels M (2016). A general framework for considering selection bias in EHR-based studies: what data are observed and why?. EGEMS (Wash DC).

[23] Grobbee DE, Hoes AW, Verheij TJM, Schrijvers AJP, van Ameijden EJC, Numans ME (2005). The Utrecht health project: optimization of routine healthcare data for research. Eur J Epidemiol.

[24] Homburg M, Berger M, Berends M (2024). Dutch GP healthcare consumption in COVID-19 heterogeneous regions: an interregional time-series approach in 2020-2021. BJGP Open.

[25] Bos I, Bosman L, van den Hoek R (2025). Comparison of observational methods to identify and characterize post-COVID syndrome in the Netherlands using electronic health records and questionnaires. PLoS ONE.

[26] Blanker MH (2023). General practice research infrastructure pandemic preparedness program (GRIP3). COVID-19 2021.

[27] Homburg M, Meijer E, Berends M (2023). A natural language processing model for COVID-19 detection based on Dutch general practice electronic health records by using bidirectional encoder representations from transformers: development and validation study. J Med Internet Res.

[28] Twickler R, Berger MY, Groenhof F (2024). Data resource profile: registry of electronic health records of general practices in the north of the Netherlands (AHON). Int J Epidemiol.

[29] (2024). Nivel primary care database. Nivel.

[30] Soler JK, Okkes I, Wood M, Lamberts H (2008). The coming of age of ICPC: celebrating the 21st birthday of the international classification of primary care. Fam Pract.

[31] Nahler G (2009). Dictionary of Pharmaceutical Medicine.

[32] Westerdijk M, Zuurbier J, Ludwig M, Prins S (2012). Defining care products to finance health care in the Netherlands. Eur J Health Econ.

[33] Nielen MMJ, Spronk I, Davids R (2019). Estimating morbidity rates based on routine electronic health records in primary care: observational study. JMIR Med Inform.

[34] Hek K, Ramerman L, Weesie YM (2022). Antibiotic prescribing in Dutch daytime and out-of-hours general practice during the COVID-19 pandemic: a retrospective database study. Antibiotics (Basel).

[35] (2024). Farmacotherapeutisch kompas [pharmacotherapeutic compass]. Zorginstituut Nederland.

[36] Austin PC (2009). Using the standardized difference to compare the prevalence of a binary variable between two groups in observational research. Commun Stat Simul Comput.

[37] Overbeek JA, Swart KMA, Houben E, Penning-van Beest FJA, Herings RMC (2023). Completeness and representativeness of the PHARMO general practitioner (GP) data: a comparison with national statistics. Clin Epidemiol.

[38] Wilkinson MD, Dumontier M, Aalbersberg IJJ (2016). The FAIR guiding principles for scientific data management and stewardship. Sci Data.

[39] Jacobsen A, Kaliyaperumal R, da Silva Santos LOB (2020). A generic workflow for the data FAIRification process. Data Intell.

[40] Chang E, Mostafa J (2021). The use of SNOMED CT, 2013-2020: a literature review. J Am Med Inform Assoc.

[41] Heins MJ, de Ligt KM, Verloop J, Siesling S, Korevaar JC (2022). Opportunities and obstacles in linking large health care registries: the primary secondary cancer care registry - breast cancer. BMC Med Res Methodol.

[42] Liaw ST, Taggart J, Dennis S, Yeo A (2011). Data quality and fitness for purpose of routinely collected data--a general practice case study from an electronic practice-based research network (ePBRN). AMIA Annu Symp Proc.

[43] Neiva FW, David JMN, Braga R, Campos F (2016). Towards pragmatic interoperability to support collaboration: a systematic review and mapping of the literature. Inf Softw Technol.

[44] Gini R, Schuemie M, Brown J (2016). Data extraction and management in networks of observational health care databases for scientific research: a comparison of EU-ADR, OMOP, mini-sentinel and MATRICE strategies. EGEMS (Wash DC).

[45] Kahn MG, Callahan TJ, Barnard J (2016). A harmonized data quality assessment terminology and framework for the secondary use of electronic health record data. EGEMS (Wash DC).

[46] Blacketer C, Defalco FJ, Ryan PB, Rijnbeek PR (2021). Increasing trust in real-world evidence through evaluation of observational data quality. J Am Med Inform Assoc.

[47] Dros JT, Bos I, Bennis FC (2022). Detection of primary Sjögren’s syndrome in primary care: developing a classification model with the use of routine healthcare data and machine learning. BMC Prim Care.

[48] (2022). Introducing ChatGPT. OpenAI.

